# Low-intensity pulsed ultrasound for ‘no-option’ chronic/critical limb-threatening ischaemia in a patient with Buerger disease: a case report

**DOI:** 10.1093/ehjcr/ytae246

**Published:** 2024-05-24

**Authors:** Farina Mohamad Yusoff, Masato Kajikawa, Takumi Sakamoto, Akio Tanaka, Yukihito Higashi

**Affiliations:** Department of Regenerative Medicine, Division of Radiation Medical Science, Research Institute for Radiation Biology and Medicine, Hiroshima University, 1-2-3 Kasumi, Minami-ku, Hiroshima 734-8551, Japan; Division of Regeneration and Medicine, Hiroshima University Hospital, Hiroshima, Japan; Department of Dermatology, Graduate School of Biomedical and Health Sciences, Hiroshima University, Hiroshima, Japan; Department of Dermatology, Graduate School of Biomedical and Health Sciences, Hiroshima University, Hiroshima, Japan; Department of Regenerative Medicine, Division of Radiation Medical Science, Research Institute for Radiation Biology and Medicine, Hiroshima University, 1-2-3 Kasumi, Minami-ku, Hiroshima 734-8551, Japan; Division of Regeneration and Medicine, Hiroshima University Hospital, Hiroshima, Japan

**Keywords:** Buerger disease, Therapeutic ultrasound, Limb ischaemia, Case report

## Abstract

**Background:**

Buerger disease, also known as Winiwarter–Buerger disease or thromboangiitis obliterans (TAO), is a non-specific inflammation of small- and medium-sized arteries with thrombus obliteration and without atherosclerotic changes. Patients with TAO can develop chronic limb-threatening ischaemia (CLTI) and are at risk of limb amputation despite smoking cessation and exercise therapy recommendations.

**Case summary:**

A 72-year-old Japanese man presented with painful discolouration of toes and renal impairment. He was diagnosed with Rutherford classification Stage 6 CLTI with immunoglobulin A nephropathy. He refused limb amputation. Clinical symptoms reduced after treatment with low-intensity pulsed ultrasound (LIPUS). LIPUS is a non-invasive option to alleviate peripheral arterial disease symptoms. Despite the initiation of conventional therapy measures, there was a worsening of the limb condition. The non-invasive investigational treatment option of LIPUS was initiated after the poor clinical outcomes of the conventional therapy measures. The patient’s symptoms in the bilateral lower limbs, ulcers, and the blue-coloured toes gradually lessened. After 1 year of treatment with LIPUS, he had achieved better walking independence with improved quality of life.

**Discussion:**

Low-intensity pulsed ultrasound is a non-invasive option for therapeutic angiogenesis with the potential to improve ischaemic limb conditions in patients with peripheral arterial disease and to avoid major amputation procedures.

**Learning points**

Case: A patient who presented with Rutherford classification Stage 6 chronic limb-threatening ischaemia (CLTI) and renal impairment.

- To be able to provide a therapeutic angiogenesis option for no-option CLTI.
- To understand the potential of regenerative medicine therapeutic modalities of low-intensity pulsed ultrasound as one of the treatment strategies for vascular diseases.

## Introduction

Buerger disease, also known as Winiwarter–Buerger disease or thromboangiitis obliterans (TAO), is a non-specific inflammation of small- and medium-sized arteries with thrombus obliteration and without atherosclerotic changes.^1–3^ Thromboangiitis obliterans affects young and middle-aged smokers, predominantly males. Patients with TAO can develop chronic limb-threatening ischaemia (CLTI) and are at risk of limb amputation despite smoking cessation and exercise therapy recommendations. Rutherford classification Stage 0 (asymptomatic) to Stage 6 (severe ischaemia ulcers or frank gangrene) has been used to describe the stages of peripheral artery disease (PAD). The clinical course of the disease varies in individuals, and the results of current conventional revascularization treatment options remain unpredictable due to unsuitable target vessels, limited native conduits, and migratory thrombophlebitis.^4,5^ In this study, we report a patient with TAO with Rutherford classification Stage 6 CLTI and localized infections who refused amputation and was provided with an option of non-invasive therapeutic angiogenesis treatment to treat his symptoms and to improve his quality of life.

## Summary figure

**Figure:**
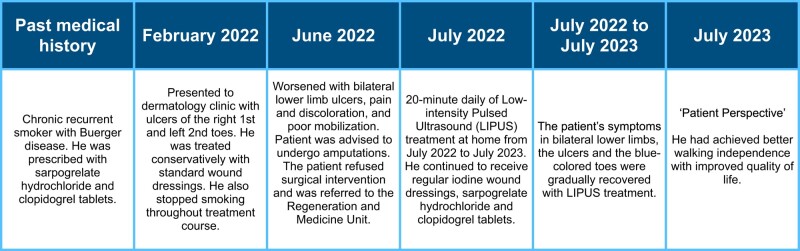


## Case presentation

**Figure 1:**
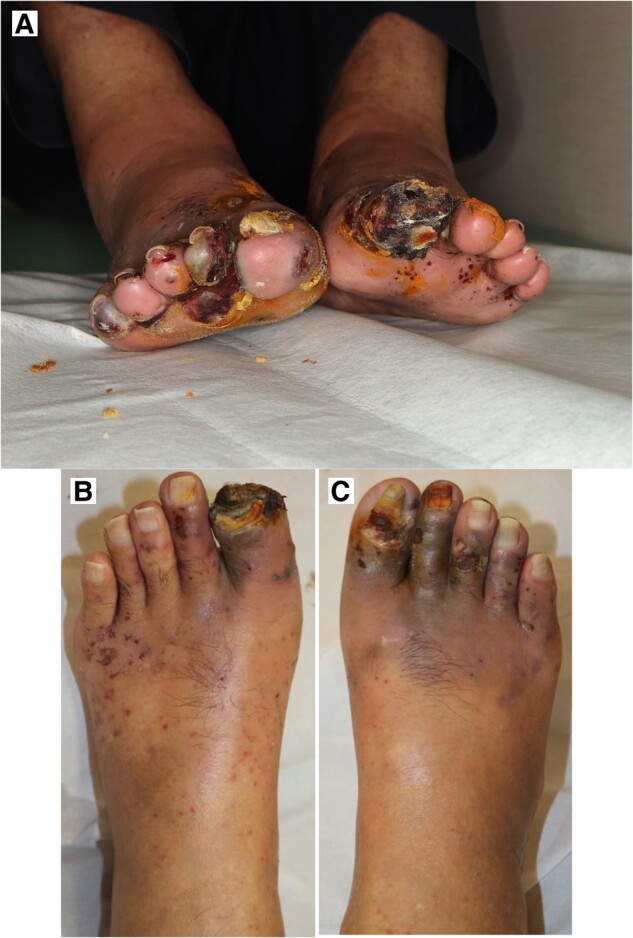
Images of the patient’s lower limbs before low-intensity pulsed ultrasound treatment (*A–C*). The ankle-brachial indexes were 1.14 on the right limb and 0.91 on the left limb with a visual analogue scale of 85.

A 72-year-old Japanese man, a chronic recurrent smoker, who was previously diagnosed with Buerger disease, presented to our dermatology clinic in February 2022 with ulcers of the right first and left second toes. There were small ulcers on the toes, and he was treated conservatively with standard wound dressings. In June 2022, his lower limb condition worsened with bilateral lower limb pain and discolouration. The patient complained of poor mobilization due to a worsening of lower limb conditions. Cyanotic lesions and multiple ulcers were found on the toes and distal phalanges (*Figure 1A–C*). He was previously diagnosed with Buerger disease and he presented to Hiroshima University Hospital with recurrent ulcers since 2011. He was prescribed with sarpogrelate hydrochloride, a selective 5-hydroxytryptamine (2A) antagonist, and clopidogrel tablets.

He was alert, afebrile, and his blood pressure was slightly elevated (130/52 mmHg) with a normal pulse rate (60 b.p.m.). He had adequate cardiac function and no indication of heart disease. The ankle-brachial indexes (ABIs) were 1.14 on the right limb and 0.91 on the left limb, with a visual analogue scale of 85. Hypoperfusion at both below-ankle regions was found using a SensiLase PAD3000 flowmeter skin perfusion pressure assessment device. The other clinical examination was unremarkable. Blood investigation revealed mild anaemia with thrombocytopenia and abnormal serum creatinine and estimated glomerular filtration rate (e-GFR) levels (*Table 1*). A renal impairment with nephropathy was suspected. Rheumatoid factor, lupus anticoagulants, and serological investigations were later assessed to rule out other vasculitis and hypercoagulable states. Renal biopsy was performed to confirm the diagnosis of immunoglobulin A (IgA) nephropathy. In view of Rutherford classification Stage 6 CLTI and localized infections, the patient was advised to undergo amputations. The patient refused surgical intervention and was referred to the Regeneration and Medicine Unit of Hiroshima University Hospital for a treatment option.

**Table 1:** Laboratory data before low-intensity pulsed ultrasound treatment in 2022

Blood count	
White blood cells, WBC (×10^4^/μL)	6.28
Red blood cells (×10^4^/μL)	3.79
Haemoglobin (g/dL)	12.1
Platelets (×10^4^/μL)	147
Biochemical test	
Blood urea nitrogen (mg/L)	14.8
Creatinine (mg/L)	0.80
e-GFR (mL/min)	72
Natrium (mEq/L)	138
Potassium (mEq/L)	4.5
C-reactive protein (mg/dL)	6.72
Glucose (mg/L)	99
Haemoglobin A1c (%)	5.9
Total protein (g/L)	7.2
Albumin (g/L)	3.6
Total bilirubin (µmol/L)	0.4
Aspartate aminotransferase (U/L)	13
Alanine transaminase (U/L)	13
Alkaline phosphatase (IU/L)	77
Triglycerides (mg/L)	71
HDL cholesterol (mg/L)	41
LDL cholesterol (mg/L)	58
Serological test	
Rheumatoid factor (U/mL)	<6.0
Anti-nuclear antibody (IU/mL)	10.0
Immunoglobulin G (mg/dL)	1416
IgA (mg/dL)	416
Immunoglobulin M (mg/dL)	173
Proteinase 3-anti-neutrophil cytoplasmic antibody (IU/mL)	<1.0
Myeloperoxidase anti-neutrophil cytoplasmic antibody (IU/mL)	<1.0
Urinalysis	
pH	6.5
Protein (g/gCr)	30 (1+)
Blood	2+
WBC	ND
Glucose	ND

ND, not detected.

**Figure 2:**
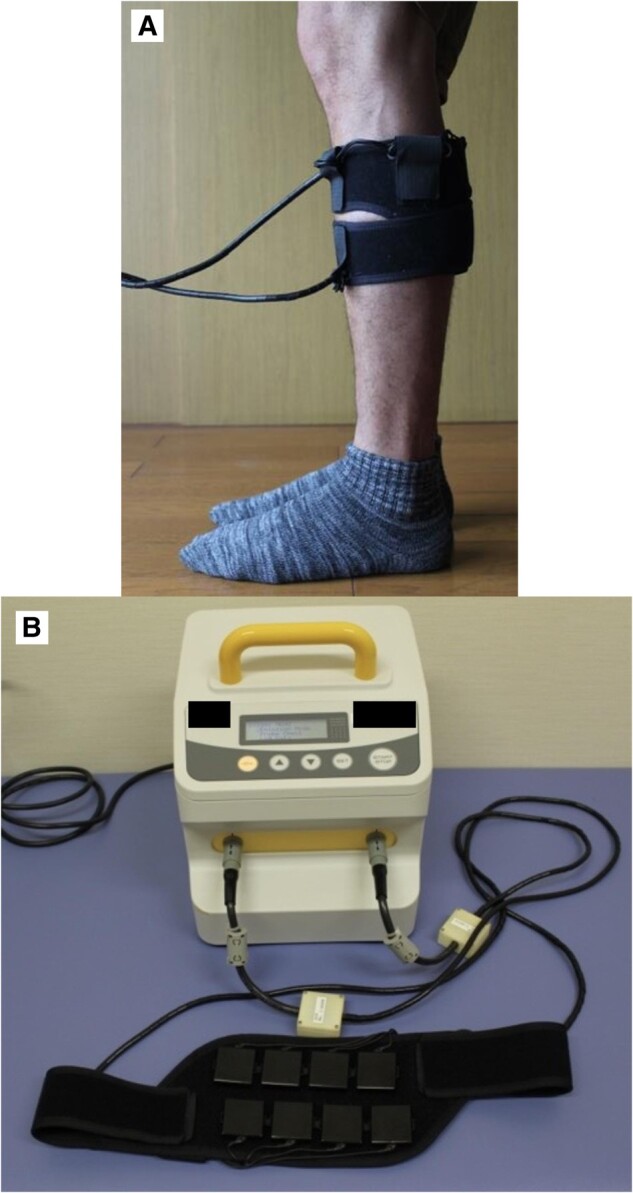
Low-intensity pulsed ultrasound. The application of the low-intensity pulsed ultrasound device on a model’s leg (*A*) and the low-intensity pulsed ultrasound device set (*B*).

The non-invasive investigational treatment option of low-intensity pulsed ultrasound (LIPUS; SX-1001, Nippon Sigmax Co., Ltd, Japan; *Figure 2*) was initiated, and the patient was instructed on how to operate the LIPUS device at home and how to apply transducers for LIPUS irradiation in the gastrocnemius of the ischaemic leg. Written informed consent was obtained from the patient. He was treated regularly with iodine wound dressings, sarpogrelate hydrochloride tablets (300 mg), clopidogrel tablets (75 mg), and 20 min daily LIPUS treatment from July 2022 to July 2023. He also stopped smoking throughout the treatment course. His compliance with LIPUS treatment and conservative therapy, including discontinued smoking, was monitored during the observed time frame.

**Figure 3:**
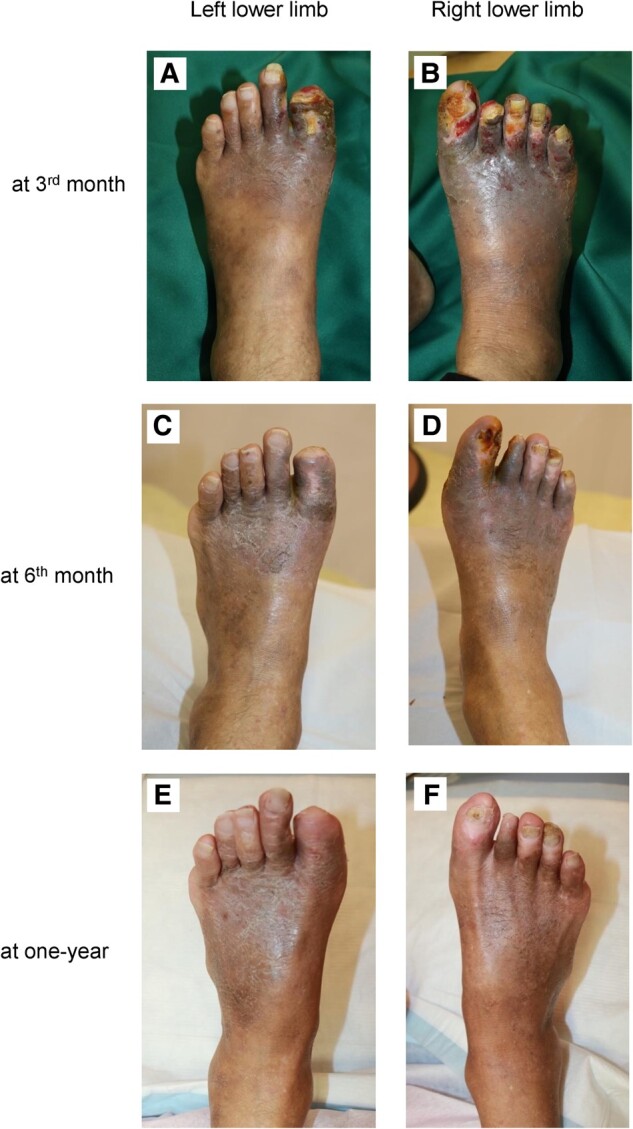
Images of the patient’s feet during ongoing treatment with low-intensity pulsed ultrasound at the 3rd month (*A*, left limb and *B*, right limb) with a visual analogue scale of 75, at the 6th month (*C*, left limb and *D*, right limb) with a visual analogue scale of 40, and at 1 year (*E*, left limb and *F*, right limb) with a visual analogue scale of 0.

During the continuous LIPUS treatment and regular wound managements, the patient’s symptoms in the bilateral lower limbs, ulcers, and the blue-coloured toes gradually reduced (*Figure 3A–F*). The ABIs at the sixth month was 1.10 on the right limb and 1.02 on the left limb, and the ABIs at 1 year was 1.27 on the right limb and 1.05 on the left limb, with an improvement of hypoperfusion at both below-ankle regions.

**Figure 4:**
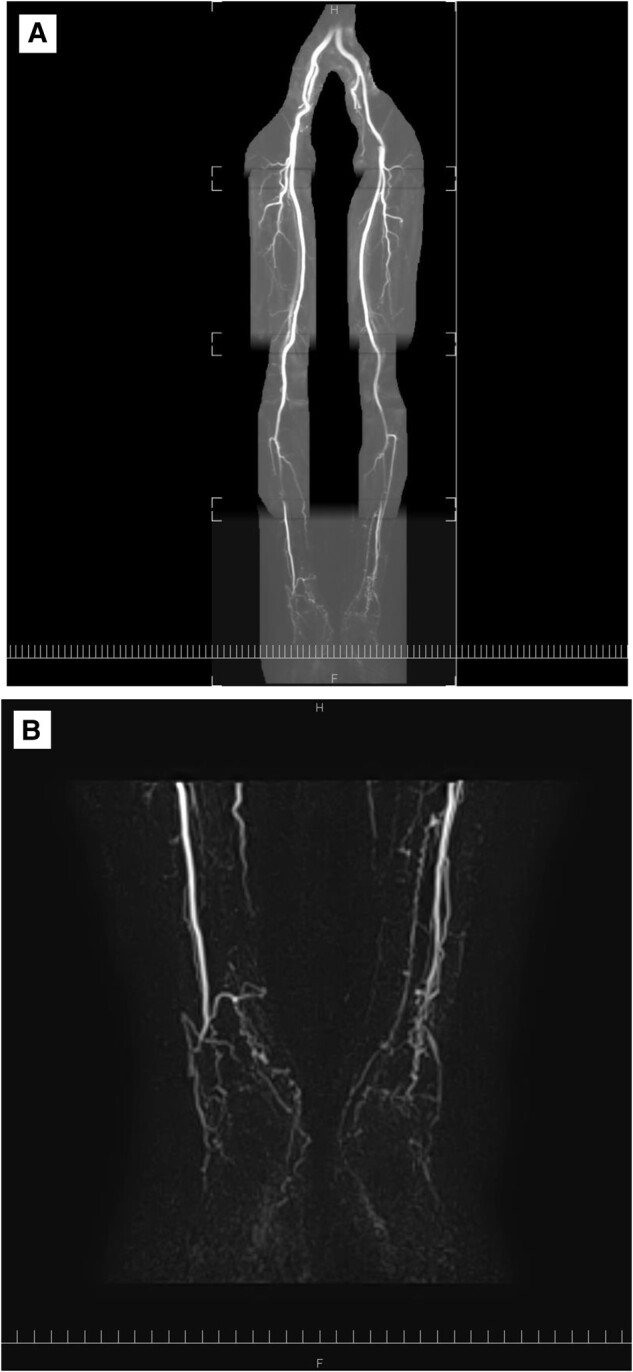
Non-contrast magnetic resonance angiography after 1 year of low-intensity pulsed ultrasound treatment. (*A*) No disruption of blood flow is seen within the imaging range. (*B*) Tortuosity and irregularity of blood vessels are noticeable in both feet.

A non-contrast magnetic resonance angiography examination was performed after 1 year of treatment with LIPUS. The results showed no disruption of blood flow within the imaging range (*Figure 4A*). The signal of both peroneal and popliteal arteries was slightly reduced, but the periphery was visualized. Tortuosity and irregularity of blood vessels were noticeable in both feet (*Figure 4B*). Throughout the period of regular follow-up, the patient reported that he adhered to smoking cessation and complied with the treatment regime, following which his walking performance improved with a reduction of his limb pain. His walking performance was evaluated by measuring maximal walking distance/time and/or pain-free walking distance/time. After 1 year of treatment with LIPUS, he had achieved better walking independence with improved quality of life.

## Discussion

This patient, who was a smoker, was diagnosed with Buerger disease in 2012 and he had recurrent chronic ulcers in the upper and lower limbs, usually after he returned to his smoking habit. Repeated therapeutic angiogenesis treatment options of cell therapy and LIPUS were provided, and clinical outcomes were satisfactory. In the most recent episode, he developed severe bilateral limb ischaemia with renal impairment. A diagnosis of IgA nephropathy, also known as Buerger disease, was previously reported in a patient with TAO.^6,7^ Circulating antibodies to collagen that are present in the blood of some patients with Buerger disease may cause an increase in cellular sensitivity to human Type I or Type III collagen or both. Renal impairment occurred in our patient during the most recent episode of CLTI. Therefore, the severity of CLTI presentation may be associated with the onset of IgA nephropathy.

Conservative therapy wound dressings, clopidogrel, sarpogrelate hydrochloride, and discontinuation of smoking were prescribed in February 2022. Despite the conventional therapy measures, there was a worsening of his limb condition in June 2022. There is no conventional revascularization treatment option for microvasculature vasculitis because of the pathological nature of the disease, which leaves patients at a high risk of amputation. The non-invasive investigational treatment option of LIPUS was initiated in July 2022 after the poor clinical outcomes of the conventional therapy measures. No adverse effects of LIPUS treatment were reported. However, we cannot deny the possibility that treatment with conservative therapy wound dressings, clopidogrel, sarpogrelate hydrochloride, and discontinuation of smoking contributed to the clinical improvement of the patient.

‘No-option’ patients with CLTI are in need of an innovative solution. LIPUS has been investigated as a treatment option for patients with PAD.^8–10^ LIPUS has been found to induce endothelial cell proliferation, migration, and sprouting with increased capillary density and increase blood flow in LIPUS-treated ischaemic hind-limb pre-clinical studies, and it has been shown that LIPUS exposure induces angiogenesis.^8,11–13^ Through a stimulation of angiogenic factors such as interleukin-8, basic fibroblast growth factor, and vascular endothelial growth factor (VEGF), via the extracellular signal-regulated kinase/Akt/endothelial nitric oxide synthase/VEGF pathway, LIPUS exposure has the potential to provide beneficial cellular therapeutic effects on limb ischaemia by inducing microvascular regeneration and to mitigate clinical symptoms in patients with CLTI and reduce the risk of amputation.^10^ Landry *et al*.^14^ reported that therapeutic ultrasound is a potential non-invasive treatment for intermittent claudication in patients with PAD, and Labeeb *et al*.^15^ found that the use of daily LIPUS with repeated intra-articular methotrexate can give better results in rheumatoid patients with resistant monoarthritis or oligoarthritis, with less side effects and without the need of adding other interventions. The mechanisms of LIPUS-induced angiogenesis are similar to those of exercise-induced angiogenesis through paracrine and metabolic effects as well as anti-inflammatory effects. LIPUS exposure increases the microvasculature in critical and chronic limb ischaemia. The prescribed daily application of LIPUS irradiation in the gastrocnemius of the ischaemic leg promotes tissue healing progression (*Figure 3A–F*), lessens clinical symptoms, and prevents limb amputation in this patient.

## Conclusion

Low-intensity pulsed ultrasound is a non-invasive option for therapeutic angiogenesis with the potential to improve ischaemic limb conditions in patients with PAD and to avoid major amputation procedures.

## Data Availability

The data underlying this article are available in the article.

## Lead author biography

Farina Mohamad Yusoff, MBBS, PhD, FSVM, is an assistant professor at the Department of Regenerative Medicine, Division of Radiation Medical Science, Research Institute for Radiation Biology and Medicine, Hiroshima University, Japan. She has a significant amount of experience in basic/fundamental, translational, and clinical research in the areas of microvascular regeneration, endothelial cell, oxidative stress, and cardiovascular effects in radiation biology, vascular medicine, and regenerative medicine.

## Notes

**Consent:** The patient consented to the publication of this case report in compliance with COPE guidelines.

**Funding:** None declared.
